# Magneto-Thermo-Elastic Theoretical Solution for Functionally Graded Thick-Walled Tube under Magnetic, Thermal and Mechanical Loads Based on Voigt Method

**DOI:** 10.3390/ma15186345

**Published:** 2022-09-13

**Authors:** Tiane Li, Jiabao Li, Xuekang Liu, Yaozhi Luo

**Affiliations:** 1 College of Civil Engineering and Architecture, Zhejiang University, Hangzhou 310058, China; 2 College of Civil Engineering, Taiyuan University of Technology, Taiyuan 030024, China; 3 Zhejiang Provincial Key Laboratory of Space Structures, Hangzhou 310058, China

**Keywords:** functionally graded materials (FGMs), thick-walled tube, magneto-thermo-elastic theoretical solution, magnetic field, mechanical loads, thermal loads

## Abstract

In this study, the mechanical responses of a functionally graded thick-walled tube simultaneously under magnetic, thermal and mechanical loads are studied. Based on the assumption that the volume fraction of each phase material is distributed as a power function, the Voigt method is used to obtain the stress–strain relationship of the functionally graded materials (FGMs). The influences of the relevant material parameters including volume fraction, thermal expansion coefficient, and Poisson’s ratio on the magneto-thermo-elastic theoretical solution are deeply studied and discussed. Furthermore, when some of the parameters are set as special values, the research results can be degenerated to two coupled loads which are consistent with the existing researches. The results of this paper provide theoretical support for the practical design and application of the FGM tube under the combined action of magnetic, thermal and mechanical loads.

## 1. Introduction

Functionally graded materials (FGMs) [1,2,3] are a type of newly developed composite material with continuous material and mechanical properties, which effectively eliminate stress concentrations in common laminates. FGMs can be designed in various shapes as needed, such as disc [4], plate [5,6,7], beam [8] and cylinder [9], etc. As a commonly used structure in engineering, functionally graded thick-walled tubes have attracted extensive attention and research in the materials industry due to their unique physical and mechanical properties, and have begun to be applied in many different practical engineering fields, such as aerospace, magnetic storage components, magnetic structural components, etc.

The magneto-thermo-elastic environment is a fairly common phenomenon in industrial applications, such as in nuclear devices [10], development of a highly sensitive superconducting magnetometer, electrical equipment [11] and optics, etc. [12]. In the last couple of decades, a number of researchers have investigated this problem.

The researches on theoretical solutions for the uniform circular tube with mechanical loads, thermal loads and magnetic fields are relatively mature [13,14,15]. In recent years, some scholars have studied theoretical solutions for the graded thick-walled tube, based on certain assumptions (see Table 1).

When the responses of the functionally graded tube under the combination of the above three loads are investigated, it is necessary to make assumptions about all the above parameters [16,17,18,19,20,21,22,23,24,25,26,27,28]. In past studies, Poisson’s ratio is set as a constant and most material parameters are assumed as power functions, such as elastic modulus *E*(*r*) = *E*_0_*r*^n^, thermal expansion coefficient *α*(*r*) = *α*_0_*r*^n^, thermal conductivity *k*(*r*) = *k*_0_*r*^n^, and magnetic permeability *μ*(*r*) = *μ*_0_*r*^n^. Some researchers also assume these material parameters as exponential functions [27], such as *E*(*r*) = *E*_0_*e*^rn^, *α*(*r*) = *α*_0_*e*^m^, *k*(*r*) = *k*_0_*e*^rn^, *μ*(*r*) = *μ*_0_ *e*^rn^ (*E*_0_, *α*_0_, *k*_0_, *μ*_0_, *m* and *n* are the material constants, and *r* is the radial coordinate).

A summary of the existing literature finds that: (1) the indexes *m* or *n* are basically the same, but this is inconsistent with objective reality if all material parameters have the same form. Some authors [28] have proposed that the indexes should not be the same. However, it is also considered the same in ultimate numerical analysis because the problems are complicated by different indexes. (2) As is well known, the elastic modulus and Poisson’s ratio are basic material parameters of the uniform isotropic materials. Therefore, it is obviously more reasonable to simultaneously make a hypothesis of elastic modulus and Poisson’s ratio when assuming the entire material parameters for a functionally graded tube. However, most scholars think that Poisson’s ratio of each layer of the tube has a small effect on the responses of the whole tube, and set Poisson’s ratio as a constant to avoid the complexity of solving equilibrium equations. Only a few scholars think that Poisson’s ratio should also be considered as another material parameter [29,30,31]. If Poisson’s ratio is also assumed as a specific function, often along with complex equilibrium equations, it is difficult to obtain theoretical solutions. (3) From the aspect of the production of engineering materials, it is difficult to change material parameters according to a certain rule. The FGMs are achieved by combining two or more homogeneous materials in a controlled proportion; this does not conform to the material’s practical manufacturing method if one simply assumes these material parameters.

In order to overcome the above problems, the equivalent material parameters of the thick-walled tube are given out using the Voigt method, based on the volume fractions and respective material parameters of the two-phase materials that compose the tube instead of assuming the material parameters. Based on this method, the existing literature [32,33,34] has given theoretical solutions of thick-walled tubes under a single load or two loads acting together. In this topic, thick-walled tubes simultaneously under magnetic, thermal and mechanical loads are studied, and the results are compared with previous literature [32,33,34] when some parameters are set as special values.

## 2. Material Models and Properties

**Figure 1:**
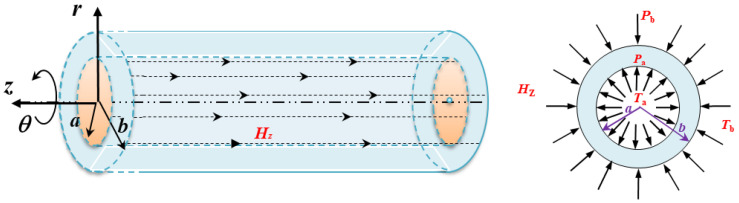
A diagram of a long FGM tube subjected to mechanical loads, thermal loads and magnetic field.

The infinitely long FGM thick-walled tube under uniform magnetic fields *H*_z_ is subjected to mechanical loads *P* and thermal loads *T*, as shown in Figure 1. Cylindrical polar coordinates (*r*, *θ*, *z*) are used, and the inner and outer radii are designated as *a* and *b*, respectively. *P*_a_ and *T*_a_ are the loads acting on the inner surface, and *P*_b_ and *T*_b_ are the loads acting on the outer surface.

The FGM thick-walled tube is composed of two distinct materials that are isotropic linear elastic Material A and linear elastic Material B. The interaction between the two materials is not considered. The volume fraction *c*(*r*) of Material A is described according to the power law [34].

(1) $$c(r)=c_{0}\left[1-k{\left(r/b\right)}^{n}\right]$$

where, $c_{0}$, *k* and *n* are the material parameters, *r* is the radius. Different distribution rules can be obtained by adjusting the material parameters, and the value of *c*(*r*) stays between 0 and 1.

## 3. Magneto-Thermo-Elastic Theoretical Solution

The relevant literature has presented the thermo-elastic theoretical solution [32] and magneto-elastic theoretical solution [33] of the thick-wall tube. In this paper, the magneto-thermo-elastic theoretical solution is further deduced on the basis of the above researches using the Voigt method, which considers that the strain of each phase material is equal and the stress is not equal when different phase materials come into contact with each other. The Voigt method can avoid making assumptions about parameters such as elastic modulus, Poisson’s ratio, thermal conductivity, thermal expansion coefficient, and magnetic permeability, and can obtain equivalent material parameters of FGMs that are more in accordance with the actual situation.

The stresses of the FGM thick-walled tube under thermal and mechanical loads are given as [32]

(2) $$\begin{array}{l} \sigma_{r}=\bar{\lambda }\frac{u}{r}+\left(\bar{\lambda }+2\bar{G}\right)\frac{du}{dr}-\left[c(r)\alpha_{0}(3\lambda_{0}+2\mu_{0})+\left(1-c(r)\right)\alpha_{1}(3\lambda_{1}+2\mu_{1})\right]T(r)\\ \sigma_{\theta }=\left(\bar{\lambda }+2\bar{G}\right)\frac{u}{r}+\bar{\lambda }\frac{du}{dr}-\left[c(r)\alpha_{0}(3\lambda_{0}+2\mu_{0})+\left(1-c(r)\right)\alpha_{1}(3\lambda_{1}+2\mu_{1})\right]T(r)\\ \sigma_{z}=\bar{\lambda }\left(\frac{u}{r}+\frac{du}{dr}\right)-\left[c(r)\alpha_{0}(3\lambda_{0}+2\mu_{0})+\left(1-c(r)\right)\alpha_{1}(3\lambda_{1}+2\mu_{1})\right]T(r) \end{array}$$

where, the superscript *i* = 0, 1, respectively, correspond to Materials A and B; $\lambda_{i}$ and $G_{i}$ are Lamé constants; the subscript *r*, *θ*, *z* represent radial, circumferential and axial direction, respectively; $\alpha_{i}$ is the thermal expansion coefficient, *u* is the displacement in radial direction; $\bar{\lambda }$ and $\bar{G}$ are defined as

(3) $$\begin{array}{l} \bar{\lambda }=c(r)\lambda_{0}+\left[1-c(r)\right]\lambda_{1}\\ \bar{G}=c(r)G_{0}+\left[1-c(r)\right]G_{1} \end{array}$$

The following assumptions are made: (a) each material component of the FGM tube is non-ferromagnetic and non-ferroelectric; (b) the Thompson effects are ignored; (c) the displacement currents are ignored; the radial Lorentz’s stress of the thick-walled tube subjected to magnetic field *H_z_* is obtained based on consideration of Maxwell’s equations [33], described as

(4) $$f_{r}=H_{z}^{2}\mu (r)\left(\frac{\partial^{2}u}{\partial r^{2}}+\frac{1}{r}\frac{\partial u}{\partial r}-\frac{u}{r^{2}}\right)$$

where

(5) $$\mu (r)=c(r)\mu_{1}+\left[1-c(r)\right]\mu_{2}$$

Substituting Equations (2) and (4) into the equilibrium equation of tube subjected to a magnetic field in the cylindrical coordinate system $\frac{d\sigma_{r}}{dr}+\frac{\sigma_{r}-\sigma_{\theta }}{r}+f_{r}=0$ [33], the ordinary differential equation for displacement can be induced as

(6) $$r\left(\phi_{1}-\phi_{2}r^{n}\right)\frac{d^{2}u}{dr^{2}}+\left(\phi_{1}-\phi_{3}r^{n}\right)\frac{du}{dr}-\left(\phi_{1}+\phi_{4}r^{n}\right)\frac{u}{r}=f(r)$$

where

(7) $$\begin{array}{l} \phi_{1}=c_{0}\left(\lambda_{1}+2G_{1}+\mu_{1}H_{z}^{2}\right)+(1-c_{0})\left(\lambda_{2}+2G_{2}+\mu_{2}H_{z}^{2}\right)\\ \phi_{2}=c_{0}k\left(\lambda_{1}+2G_{1}+\mu_{1}H_{z}^{2}-\lambda_{2}-2G_{2}-\mu_{2}H_{z}^{2}\right)/b^{n}\\ \phi_{3}=(n+1)\phi_{2}-c_{0}knH_{z}^{2}(\mu_{1}-\mu_{2})/b^{n}\\ \phi_{4}=c_{0}kn(\lambda_{1}-\lambda_{2})/b^{n}-\phi_{2}\\ f(r)=Anr^{n}\ln r+B+Cr^{2n}+Dr^{n} \end{array}$$

and constants in f(r) are

(8) $$\begin{array}{l} A=\frac{c_{0}C_{1}k}{b^{n}}\left[\alpha_{1}\left(3\lambda_{1}+2\mu_{1}\right)-\alpha_{0}\left(3\lambda_{0}+2\mu_{0}\right)\right]\\ B=C_{1}\left[\alpha_{1}\left(1-c_{0}\right)\left(3\lambda_{1}+2\mu_{1}\right)+c_{0}\alpha_{0}\left(3\lambda_{0}+2\mu_{0}\right)\right]\\ C=\frac{2\left(k_{0}-k_{1}\right)c_{0}k}{(c_{0}k_{1}+k_{0}-c_{0}k_{0})b^{n}}A\\ D=\frac{(nC_{2}+C_{1})A}{C_{1}}+\frac{CB}{2A} \end{array}$$

As is well known, $\phi_{1}$ includes parameters for Material A and Material B, which are generally not equal to zero, so Equation (6) can be transformed into

(9) $$r\left(1-\frac{\phi_{2}}{\phi_{1}}r^{n}\right)\frac{d^{2}u}{dr^{2}}+\left(1-\frac{\phi_{3}}{\phi_{1}}r^{n}\right)\frac{du}{dr}-\left(1+\frac{\phi_{4}}{\phi_{1}}r^{n}\right)\frac{u}{r}=\frac{C}{\phi_{1}}r^{2n}+\frac{nA}{\phi_{1}}r^{n}\ln r+\frac{D}{\phi_{1}}r^{n}+\frac{B}{\phi_{1}}$$

Substituting $x=\chi (r)=\frac{\phi_{2}}{\phi_{1}}r^{n}$ into Equation (9)

(10) $$x^{2}\left(1-x\right)\frac{d^{2}u}{dx^{2}}+x\left(1-\frac{n-1+\phi_{3}/\phi_{2}}{n}x\right)\frac{du}{dx}-\frac{1}{n^{2}}\left(1+\frac{\phi_{4}}{\phi_{2}}x\right)u=x^{2}\left(1-x\right)g\left(x\right)$$

where

(11) $$g(x)=\frac{{\left(\frac{\phi_{1}}{\phi_{2}}x\right)}^{1/n}\left[\frac{C\phi_{1}}{\phi_{2}}x^{2}+\frac{A}{\phi_{2}}x\ln\left(\frac{\phi_{1}}{\phi_{2}}x\right)+\frac{D}{\phi_{2}}x+\frac{B}{\phi_{1}}\right]}{n^{2}x^{2}(1-x)}$$

Equation (10) is a second-order inhomogeneous ordinary differential equations, and its solution includes two parts: the general solution $u_{c}(x)$ of the homogeneous equation and the specific solution $u_{p}(x)$ of the inhomogeneous equation. The general solution in the interval −1 < *x* < 1 can be expressed as

(12) $$u_{c}(x)=B_{1}u_{1}(x)+B_{2}u_{2}(x)=B_{1}rF(\alpha ,\beta ,\delta ;x)+B_{2}\frac{1}{r}F(\alpha -\delta +1,\beta -\delta +1,2-\delta ;x)$$

where, *B*_1_ and *B*_2_ are integration constant, *F* is the hypergeometric series, which has been defined in reference [32]. For common functionally graded material parameters, *x* is mainly concentrated in −1 < *x* < 1. For all the other *x* values, it can be converted to −1 < *x* < 1 by simple variable substitution, which will not be repeated here.

The coefficients in the hypergeometric series are

(13) $$\delta =1+\frac{2}{n},\;\alpha =\frac{\sqrt{{\left(\phi_{3}/\phi_{2}-1\right)}^{2}-4\phi_{4}/\phi_{2}}+\phi_{3}/\phi_{2}+1}{2n},\;\beta =\frac{\phi_{3}/\phi_{2}+1}{n}-\alpha$$

A specific solution of Equation (10) is easily obtained by the constant variation method.

(14) $$u_{p}(x)=-u_{1}(x)\int_{x_{a}}^{x}\frac{u_{2}(t)g(t)}{W(t)}dt+u_{2}(x)\int_{x_{a}}^{x}\frac{u_{1}(t)g(t)}{W(t)}dt$$

where, $x_{a}=\chi (a)$, and Wronskian is

(15) $$W(x)=u_{1}(x){u^{\prime }}_{2}(x)-u_{2}(x){u^{\prime }}_{1}(x)$$

Then, the solution of Equation (10) can be written as

(16) $$u(x)=u_{1}(x)\left(B_{1}-\int_{x_{a}}^{x}\frac{u_{2}(t)g(t)}{W(t)}dt\right)+u_{2}(x)\left(B_{2}+\int_{x_{a}}^{x}\frac{u_{1}(t)g(t)}{W(t)}dt\right)$$

Substituting Equation (16) into the Equation (2), stresses in all directions and the magnetic field of the FGM tube can be induced as

(17) $$\begin{array}{l} \sigma_{r}=\bar{\lambda }\frac{u(x)}{r}+\left(\bar{\lambda }+2\bar{G}\right)u^{\prime }(x)-\left[c(r)\alpha_{0}(3\lambda_{0}+2\mu_{0})+\left(1-c(r)\right)\alpha_{1}(3\lambda_{1}+2\mu_{1})\right]T(r)\\ \sigma_{\theta }=\left(\bar{\lambda }+2\bar{G}\right)\frac{u(x)}{r}+\bar{\lambda }u^{\prime }(x)-\left[c(r)\alpha_{0}(3\lambda_{0}+2\mu_{0})+\left(1-c(r)\right)\alpha_{1}(3\lambda_{1}+2\mu_{1})\right]T(r)\\ \sigma_{z}=\bar{\lambda }\left(\frac{u(x)}{r}+u^{\prime }(x)\right)-\left[c(r)\alpha_{0}(3\lambda_{0}+2\mu_{0})+\left(1-c(r)\right)\alpha_{1}(3\lambda_{1}+2\mu_{1})\right]T(r)\\ h_{z}=-H_{z}\left(\frac{u(x)}{r}+u^{\prime }(x)\right) \end{array}$$

With stress boundary conditions ${\left.\sigma_{r}\right|}_{r=a}=-p_{a}$ and ${\left.\sigma_{r}\right|}_{r=b}=-p_{b}$, the specific values of the constants *B*_1_ and *B*_2_ can be written as

(18) $$\begin{array}{l} B_{1}=-p\left[(\bar{\lambda }(b)+2\bar{G}(b))Q^{\prime }(b)+\bar{\lambda }(b)Q(b)/b\right]/B_{0}\\ B_{2}=p\left[(\bar{\lambda }(b)+2\bar{G}(b))P^{\prime }(b)+\bar{\lambda }(b)P(b)/b\right]/B_{0} \end{array}$$

where

(19) $$\begin{array}{c} B_{0}=\left[(\bar{\lambda }(b)+2\bar{G}(b))Q^{\prime }(b)+\bar{\lambda }(b)Q(b)/b\right]\left[(\bar{\lambda }(a)+2\bar{G}(a))P^{\prime }(a)+\bar{\lambda }(a)P(a)/a\right]\\ -\left[(\bar{\lambda }(a)+2\bar{G}(a))Q^{\prime }(a)+\bar{\lambda }(a)Q(a)/a\right]\left[(\bar{\lambda }(b)+2\bar{G}(b))P^{\prime }(b)+\bar{\lambda }(b)P(b)/b\right] \end{array}$$

## 4. Results and Discussion

The dimensionless representation of the radial coordinate, the inner radius, the stresses, the radial displacements and the magnetic field are $\bar{r}=r/b$, $\bar{a}=a/b$, ${\bar{\sigma }}_{ij}=\frac{\sigma_{ij}}{E_{1}\alpha_{1}f_{2}}$, $\bar{u}=\frac{uE_{1}}{p_{a}\alpha_{1}f_{2}b}$, ${\bar{h}}_{z}=\frac{h_{z}E_{1}}{H_{z}p_{a}\alpha_{1}f_{2}}$. The parameters values are taken as $\bar{a}=0.7$, $c_{0}=1$, *k* = 1, $E_{1}=70\;\mathrm{Gpa}$, $E_{0}=3E_{1}$, $\mu_{1}=\mu_{2}=4\pi \times 10^{-7}H/m$.

In the following simulations, the mechanical responses of the thick-walled tube within magnetic loads (*H*_z_ = 2.23 × 10^9^ A/m), thermal loads (*T*_a_ = 0 °C, *T*_b_ = 100 °C) and mechanical loads (*P*_a_ = 1 GPa, *P*_b_ = 0 GPa) are discussed.

### 4.1. Effects of Parameter n

The results of different parameters *n* (*n* = 1.5, 3, 5, 10) are presented to discuss the influence of the volume fraction. In this section, $v_{0}=v_{1}=0.3$, $\alpha_{1}/\alpha_{0}=2$.

**Figure 2:**
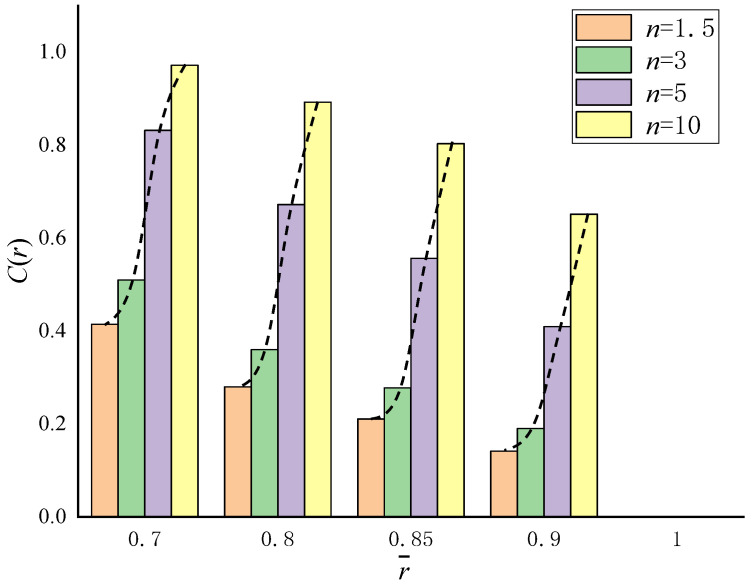
Evolution of volume fraction of Material A with different parameter *n* (*c*_0_ = 1, *k* = 1).

According to Equation (1), the volume fraction of Material A at key locations such as internal, middle and external positions are given, as shown in Figure 2. With the increase of parameter *n*, the volume fraction of Material A displays a nonlinear increasing trend. For *n* = 10, the volume fraction approaches 1.0 at the inner radius $\bar{r}=0.7$. For different parameter *n*, the volume fraction of Material A decreases from the inner radius to outer radius, and equals zero at the outer radius $\bar{r}=1.0$.

To make the results distinct and understandable, Table 2 lists the extremum values at the inner and outer radii corresponding to different parameter *n*.

**Figure 3:**
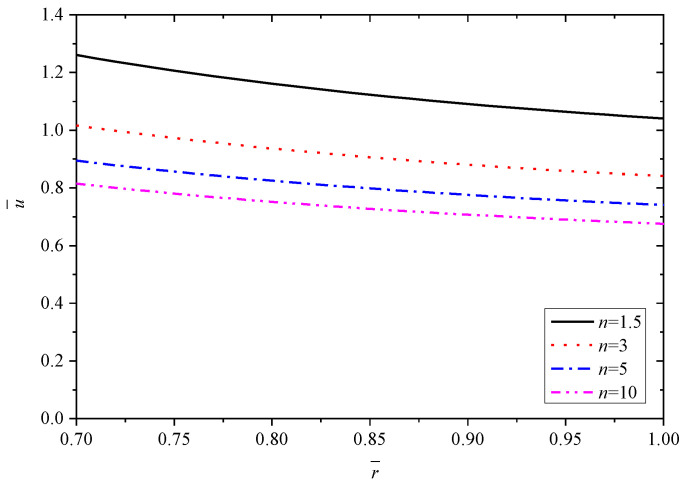
Effects of *n* on the radial displacement $\bar{u}$.

**Figure 4:**
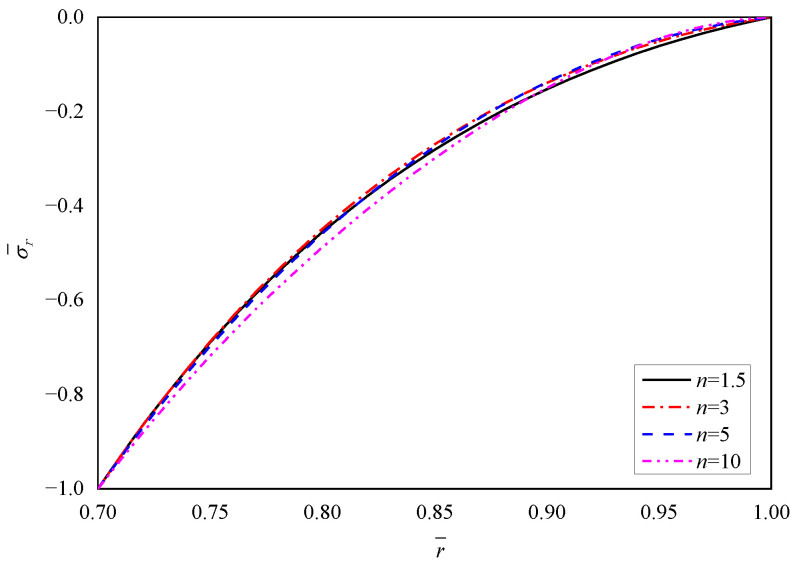
Effects of *n* on the radial stress ${\bar{\sigma }}_{r}$.

**Figure 5:**
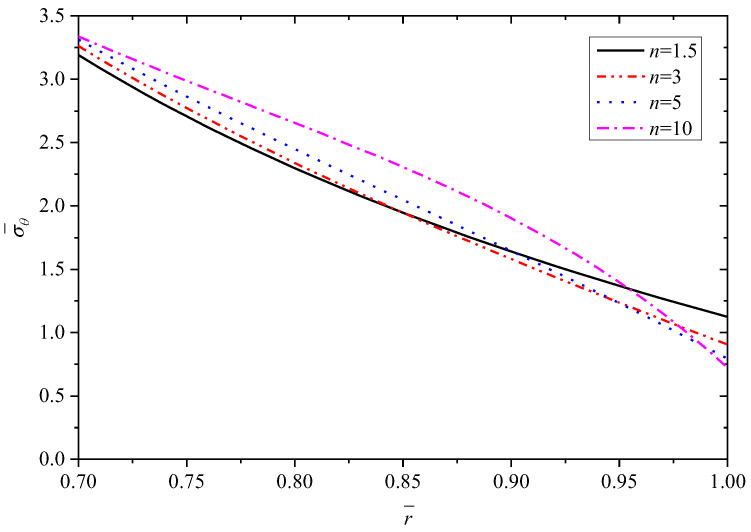
Effects of *n* on the circumferential stress ${\bar{\sigma }}_{\theta }$.

**Figure 6:**
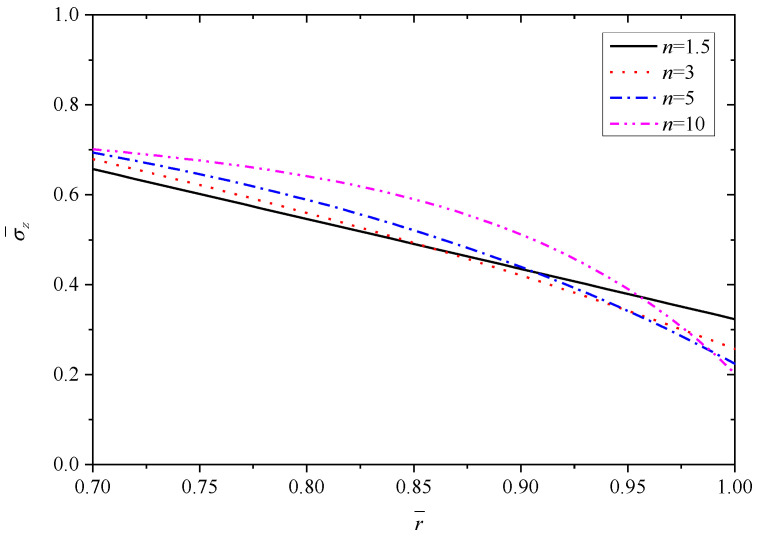
Effects of *n* on the axial stress ${\bar{\sigma }}_{z}$.

**Figure 7:**
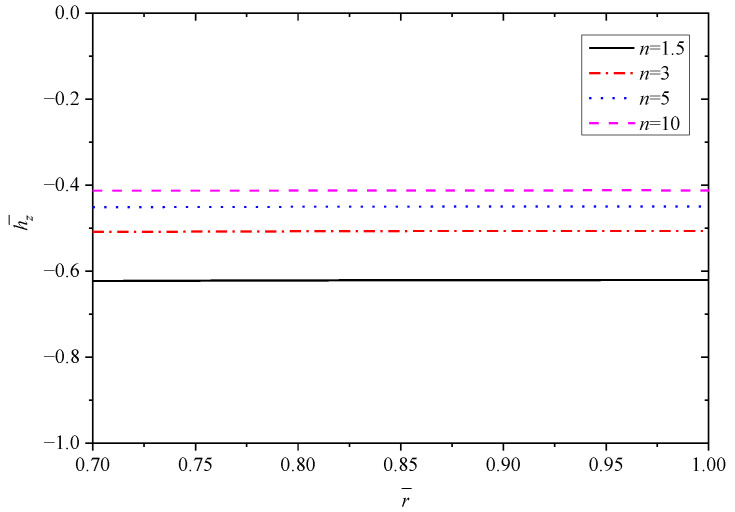
Effects of *n* on the perturbation of magnetic field ${\bar{h}}_{z}$.

Figure 3, Figure 4, Figure 5, Figure 6 and Figure 7 show the effect of parameter *n* on the radial displacement $\bar{u}$, the radial stress ${\bar{\sigma }}_{r}$, the circumferential stress ${\bar{\sigma }}_{\theta }$, the axial stress ${\bar{\sigma }}_{z}$ and the perturbation of magnetic field ${\bar{h}}_{z}$, respectively.

Figure 3 shows that parameter *n* has a marked impact on the value of radial displacement $\bar{u}$, but almost has no impact on the curve law. It is clear that the radial displacement $\bar{u}$ decreases by increasing the values of parameter *n*. The variable value of $\bar{u}$ from *n* = 1.5 to *n* = 3 is greater than that from *n* = 5 to *n* = 10, which indicates that the influence is relatively larger with smaller *n*, as listed in Table 2.

Comparing Figure 4, Figure 5 and Figure 6, it is found that the parameter *n* has significant influence on the circumferential stress ${\bar{\sigma }}_{\theta }$ and the axial stress ${\bar{\sigma }}_{z}$, but the influence on the radial stress ${\bar{\sigma }}_{r}$ can be negligible. From the inner surface to the outer surface, the radial stress ${\bar{\sigma }}_{r}$ shows the same nonlinear increasing curve shape with different parameter *n*, and all the curves obey the stress boundary condition that is $\bar{\sigma_{r}}(\bar{r})=-1$ at the inner radius ($\bar{r}=0.7$) and $\bar{\sigma_{r}}(\bar{r})=0$ at the outer radius ($\bar{r}=1.0$), as shown in Figure 4 and listed in Table 2. The stresses in other directions (${\bar{\sigma }}_{\theta }$ or ${\bar{\sigma }}_{z}$) show a downward trend from the inner radius to the outer radius, and the values exhibit fluctuations corresponding to different parameter *n* at the same radius. The curvature of curves are different and the curves intersect near the outer radius, as shown in Figure 5 and Figure 6.

Figure 7 reveals that the perturbation of magnetic field ${\bar{h}}_{z}$ increases by increasing the parameter *n*, and all the values are negative with horizontal distribution law. The value of ${\bar{h}}_{z}$ increases by about 0.11 from 1.5 to 3, and about 0.09 from 3 to 10, which indicates that the increase rate of ${\bar{h}}_{z}$ shows a downward trend as the increase of parameter *n*.

### 4.2. Effects of Thermal Expansion Coefficient

In this section, four groups of thermal expansion coefficient (*α*_1_*/α*_0_ = 0.5, 1, 2, 5) are analyzed to discuss the effects of thermal expansion coefficient. *α*_1_*/α*_0_ represents the thermal expansion coefficient ratio of Materials A and B. In this section, *n* = 1.5, $v_{0}=v_{1}=0.3$.

To make the results distinct and understandable, Table 3 lists the extremum values at the inner and outer radii corresponding to different $\alpha_{1}/\alpha_{0}$.

**Figure 8:**
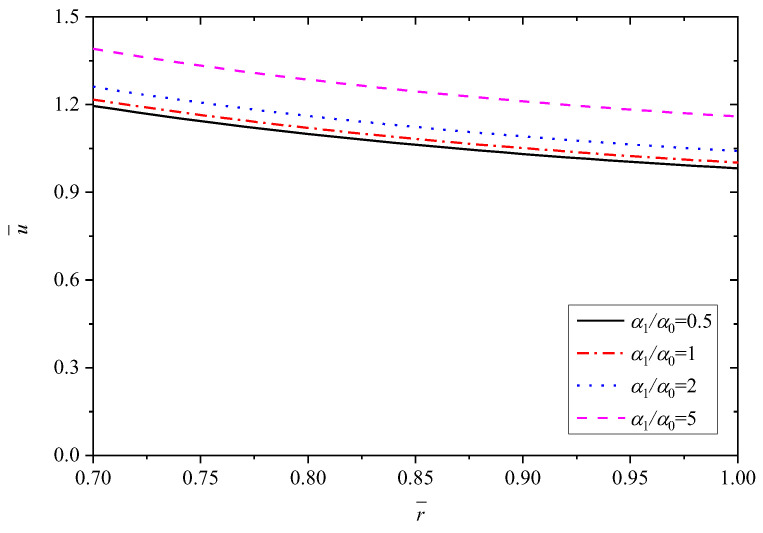
Effects of $\alpha_{1}/\alpha_{0}$ on the radial displacement $\bar{u}$.

**Figure 9:**
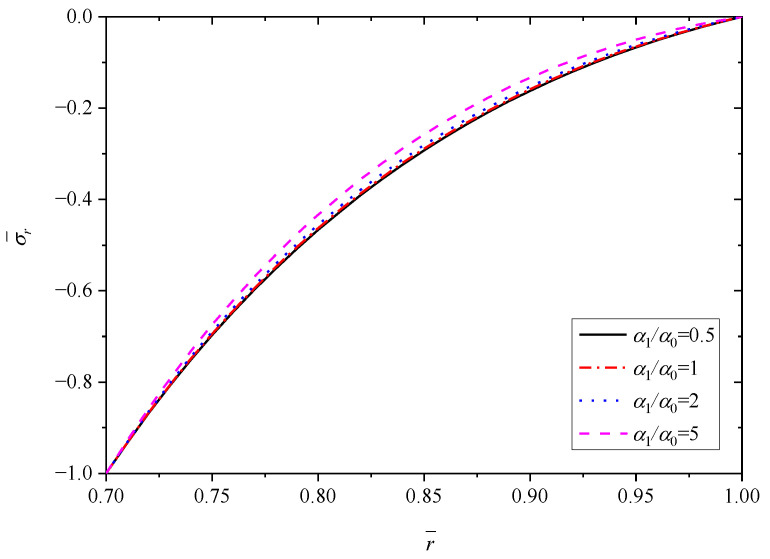
Effects of $\alpha_{1}/\alpha_{0}$ on the radial stress ${\bar{\sigma }}_{r}$.

**Figure 10:**
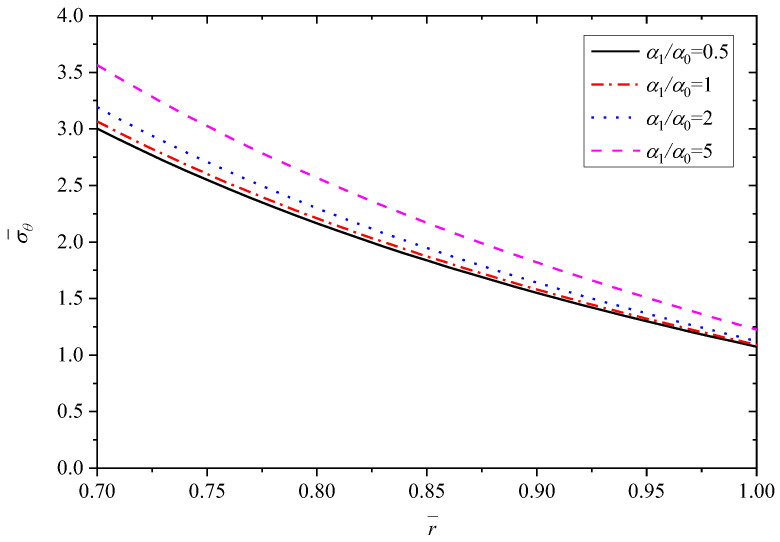
Effects of $\alpha_{1}/\alpha_{0}$ on the circumferential stress ${\bar{\sigma }}_{\theta }$.

**Figure 11:**
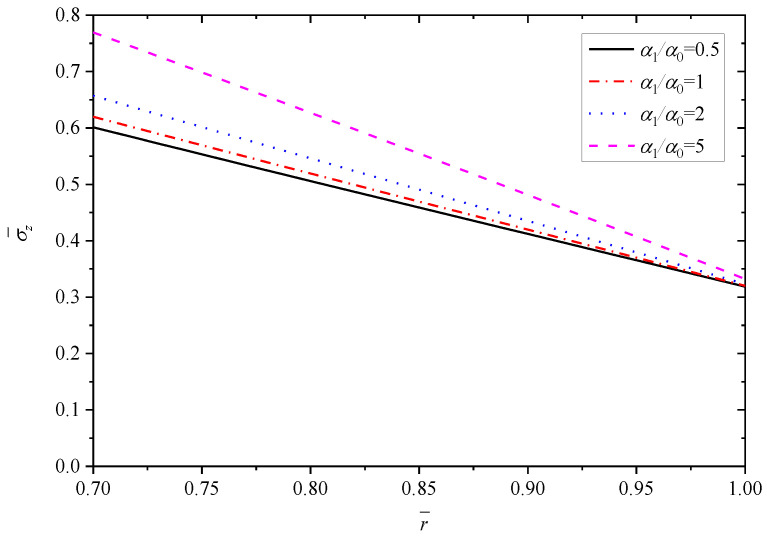
Effects of $\alpha_{1}/\alpha_{0}$ on the axial stress ${\bar{\sigma }}_{z}$.

**Figure 12:**
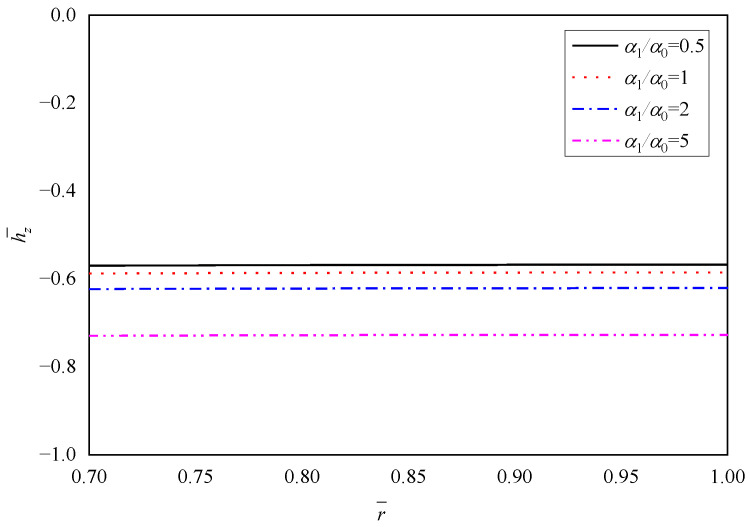
Effects of $\alpha_{1}/\alpha_{0}$ on the perturbation of magnetic field ${\bar{h}}_{z}$.

Figure 8, Figure 9, Figure 10, Figure 11 and Figure 12 show the effect of the thermal expansion coefficient on the radial displacement $\bar{u}$, the radial stress ${\bar{\sigma }}_{r}$, the circumferential stress ${\bar{\sigma }}_{\theta }$, the axial stress ${\bar{\sigma }}_{z}$ and the perturbation of magnetic field ${\bar{h}}_{z}$, respectively.

As depicted in Figure 8, the radial displacement $\bar{u}$ shows a clear increasing trend by increasing $\alpha_{1}/\alpha_{0}$, and also $\alpha_{1}/\alpha_{0}$ hardly affect the decline law of $\bar{u}$ from inner to outer radius. Different with parameter *n*, the influence on $\bar{u}$ is relatively larger with larger $\alpha_{1}/\alpha_{0}$, which is reflected by the data in Table 3.

As shown in Figure 9, similar to the effect of parameter *n* on the radial stress ${\bar{\sigma }}_{r}$, all the curves obey the stress boundary condition. From the inner radius ($\bar{r}=0.7$) to outer radius ($\bar{r}=1.0$), the biggest impact occurs at $\bar{r}=0.85$, where the radial stress ${\bar{\sigma }}_{r}$ increases along with the increase of $\alpha_{1}/\alpha_{0}$. On the whole, $\alpha_{1}/\alpha_{0}$ has minor influence on the radial stress ${\bar{\sigma }}_{r}$. Unlike the effect on radial stress ${\bar{\sigma }}_{r}$, Figure 10 and Figure 11 exhibit clearly that the effects of $\alpha_{1}/\alpha_{0}$ on the circumferential stress ${\bar{\sigma }}_{\theta }$ and axial stress ${\bar{\sigma }}_{z}$ are great. In general, the stresses ${\bar{\sigma }}_{\theta }$ (or ${\bar{\sigma }}_{z}$) show an increase trend with the increase of $\alpha_{1}/\alpha_{0}$. The influence of $\alpha_{1}/\alpha_{0}$ on the ${\bar{\sigma }}_{\theta }$ (or ${\bar{\sigma }}_{z}$) is greater for positions close to the inner radius ($\bar{r}=0.7$) than for those close to the outer radius ($\bar{r}=1.0$). For the stress ${\bar{\sigma }}_{\theta }$, the variation is about 0.56 with different $\alpha_{1}/\alpha_{0}$ at the inner radius ($\bar{r}=0.7$), and the variation is only 0.15 with different $\alpha_{1}/\alpha_{0}$ at the outer radius ($\bar{r}=1.0$), as listed in Table 3. For the stress ${\bar{\sigma }}_{z}$, the values with different $\alpha_{1}/\alpha_{0}$ at the outer radius ($\bar{r}=1.0$) are basically equal to 0.32. However, at the inner radius ($\bar{r}=0.7$), the values with different $\alpha_{1}/\alpha_{0}$ vary greatly and increase from 0.6011 to 0.7694.

The perturbation of magnetic field ${\bar{h}}_{z}$ shown in Figure 12 decreases with the increase of $\alpha_{1}/\alpha_{0}$, and all the values are negative with the horizontal distribution law.

### 4.3. Effects of Poisson’s Ratio

In this subsection, the effects of Poisson’s ratio are dicussed. Poisson’s ratio of Material A ($v_{0}$) is set as 0.3, and Poisson’s ratio of Material B ($v_{1}$) changes are 0.2, 0.3, 0.4. In this section, *n* = 1.5, $\alpha_{1}/\alpha_{0}=2$.

To make the results distinct and understandable, Table 4 lists the extremum values at the inner and outer radii corresponding to different $v_{1}$ when $v_{0}=0.3$.

**Figure 13:**
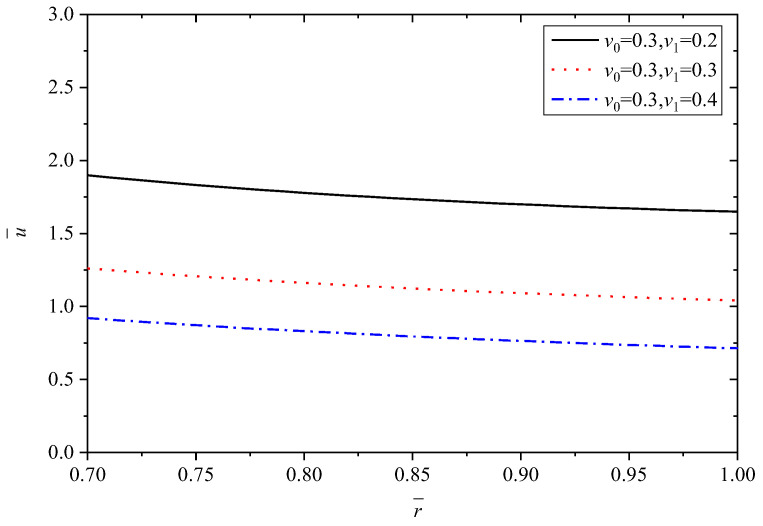
Effects of Poisson’s ratio on the radial displacement $\bar{u}$.

**Figure 14:**
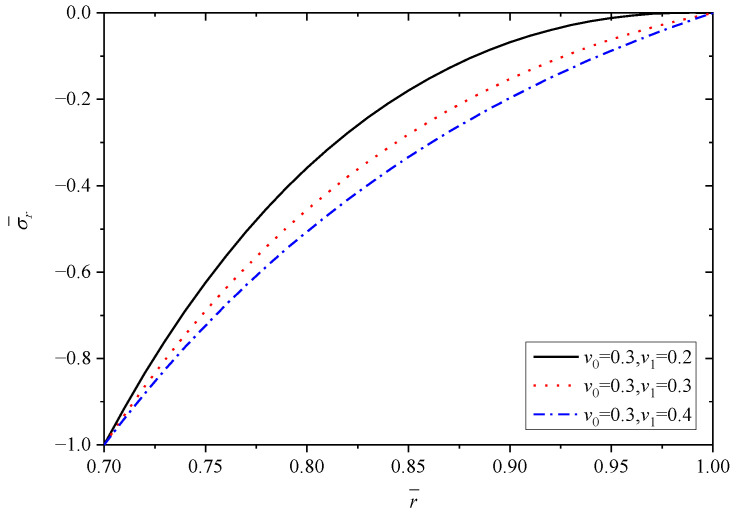
Effects of Poisson’s ratio on the radial stress ${\bar{\sigma }}_{r}$.

**Figure 15:**
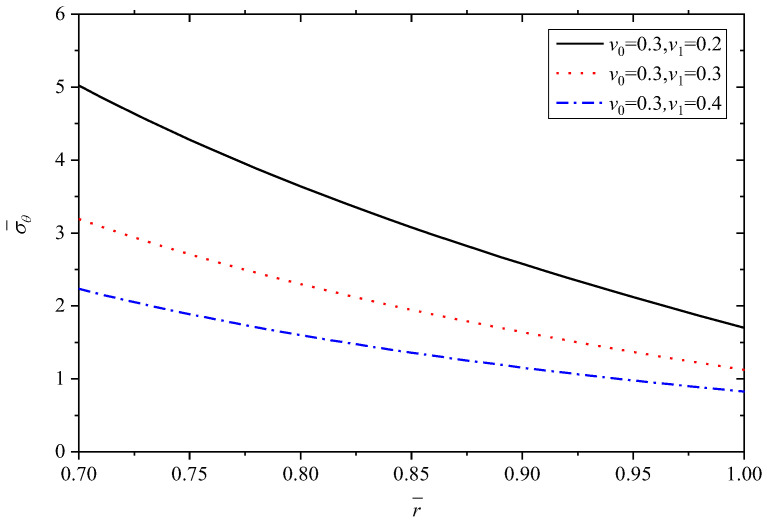
Effects of Poisson’s ratio on the circumferential stress ${\bar{\sigma }}_{\theta }$.

**Figure 16:**
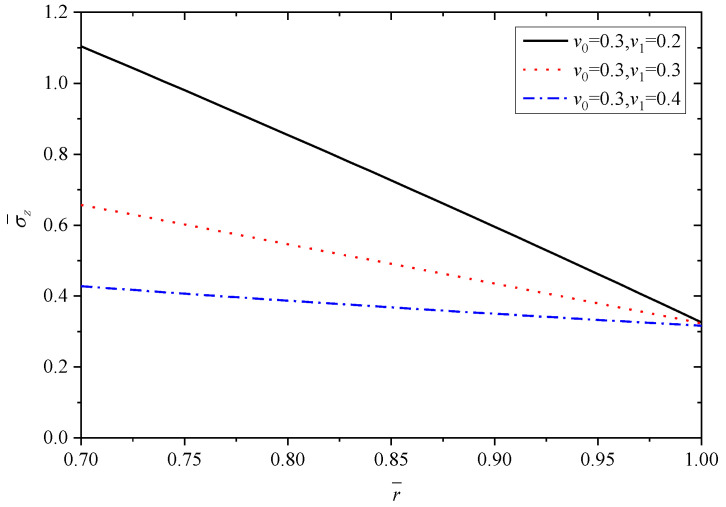
Effects of Poisson’s ratio on the axial stress ${\bar{\sigma }}_{z}$.

**Figure 17:**
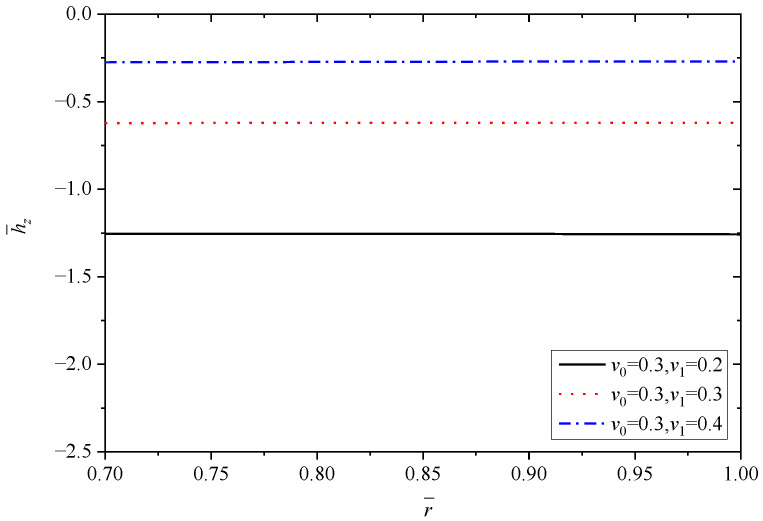
Effects of Poisson’s ratio on the perturbation of magnetic field ${\bar{h}}_{z}$.

Figure 13, Figure 14, Figure 15, Figure 16 and Figure 17 show the effect of Poisson’s ratio on the radial displacement $\bar{u}$, the radial stress ${\bar{\sigma }}_{r}$, the circumferential stress ${\bar{\sigma }}_{\theta }$, the axial stress ${\bar{\sigma }}_{z}$ and the perturbation of magnetic field ${\bar{h}}_{z}$, respectively.

The variations of radial displacement $\bar{u}$ due to Poisson’s ratio are shown in Figure 13. It is clearly found that the radial displacement $\bar{u}$ shows a significant decreasing trend along with the increase of $v_{1}$ when $v_{0}$ is set as a constant value.

Differently to the influence of the two parameters mentioned above, Poisson’s ratio displays an obvious influence on all the stresses, as shown in Figure 14, Figure 15 and Figure 16. From the curves in Figure 14, the values of radial stress ${\bar{\sigma }}_{r}$ at the inner radius and outer radius for different Poisson’s ratios are equal to −1.0 and 0.0, respectively, which indicates that all the curves obey the stress boundary condition, as listed in Table 4. However, the values from the inner radius to outer radius show significant fluctuations with the change of Poisson’s ratios, and the largest difference occurs around the middle of tube $\bar{r}=0.85$. It is obviously seen from Figure 15 and Figure 16 that Poisson’s ratio has greater effect on circumferential stress ${\bar{\sigma }}_{\theta }$ and axial stress ${\bar{\sigma }}_{z}$ near the inner radius than that close to the outer radius. Especially for the axial stress ${\bar{\sigma }}_{z}$, the same values can be obtained at the external position ($\bar{r}=1.0$) corresponding to different Poisson’s ratios, but the values change from about 0.43 to about 1.10 at the internal position ($\bar{r}=0.7$), as shown in Figure 16. Indicated in Figure 17, the increasing Poisson’s ratio $v_{1}$ results in significant change of magnetic field ${\bar{h}}_{z}$ at the same radius $\bar{r}$, and also Poisson’s ratio almost has no impact on the horizontal distribution law.

The above discussions indicate that the influence of Poisson’s ratio cannot be ignored when the analysis of functionally graded thick-walled tubes within mechanical, thermal and magnetic loads is conducted.

### 4.4. Some Special Cases

The characteristic of this paper is that the coupling effect of mechanical load, temperature load, and magnetic field are fully considered, and it has a wide range of applications; that is to say, the present work can degenerate to the same results as previous papers when some of the material parameters take special values. Based on the *n* = 1.5, $\alpha_{1}/\alpha_{0}=2$, $v_{0}=v_{1}=0.3$, the applied load values of three special cases are listed in Table 5.

For case 1, according to the theoretical formulas obtained in this paper and the theoretical formulas in reference [32], the values of material parameters at key locations such as internal ($\bar{r}=0.7$), middle ($\bar{r}=0.85$) and external ($\bar{r}=1.0$) positions are obtained. Similarly, the same operations are performed for case 2 and case 3. All the results are listed in Table 6.

By calculating, it can be found that for different cases, the theoretical solutions obtained in this paper are consistent with the values obtained by the references, as listed in Table 6. This indicates (1) the accuracy of the theoretical research in this paper; (2) the wide application range of this research, which covers the above three working conditions well.

## 5. Conclusions

The magneto-thermo-elastic theoretical solutions for an infinitely long FGM thick-walled tube composed of two materials are investigated, and the influences of the parameter *n*, the thermal expansion coefficient and Poisson’s ratio are discussed. The results indicate that all the above parameters have explicit influences on the value of radial displacement, the circumferential stress, the axial stress and the perturbation of the magnetic field. For the radial stress, all the above parameters have no effect at the inner and outer boundaries. In the middle range, the influence of Poisson’s ratio on the radial stress is significant, and the influences of the other two parameters are not significant. The curve changing laws of the radial displacement, radial stress and perturbation of magnetic field are basically not affected by all the parameters. Furthermore, by comparing the research results in this paper with the previous works when some of the parameters take special values, a more extensive scope of application is illustrated. Research in this paper can provide effective guidance for engineering design to make FGM tubes with high reliability in structural performance when subjected to a multi-field environment.

## Figures and Tables

**Table 1:** The assumptions corresponding to different problems.

Research Contents	Assumptions
response under mechanical loads	elastic modulus, Poisson’s ratio
response under thermal loads	thermal expansion coefficient, thermal conductivity
response within magnetic fields	magnetic permeability

**Table 2:** The extremum values corresponding to different parameter *n*.

*n*		1.5	3	5	10
$\bar{u}$	$\bar{r}=0.7$	1.2608	1.0163	0.8946	0.8150
	$\bar{r}=1.0$	1.0410	0.8407	0.7409	0.6756
${\bar{\sigma }}_{r}$	$\bar{r}=0.7$	−1	−1	−1	−1
	$\bar{r}=1.0$	0	0	0	0
${\bar{\sigma }}_{\theta }$	$\bar{r}=0.7$	3.1908	3.2635	3.3125	3.3376
	$\bar{r}=1.0$	1.1239	0.9039	0.7942	0.7225
${\bar{\sigma }}_{z}$	$\bar{r}=0.7$	0.6572	0.6790	0.6937	0.7012
	$\bar{r}=1.0$	0.3231	0.2571	0.2242	0.2027
${\bar{h}}_{z}$	$\bar{r}=0.7$	−0.6229	−0.5086	−0.4514	−0.4129
	$\bar{r}=1.0$	−0.6287	−0.5064	−0.4494	−0.4121

**Table 3:** The extremum values corresponding to different $\alpha_{1}/\alpha_{0}$.

$\alpha_{1}/\alpha_{0}$		0.5	1	2	5
$\bar{u}$	$\bar{r}=0.7$	1.1956	1.2173	1.2608	1.3910
	$\bar{r}=1.0$	0.9818	1.0015	1.0410	1.1593
${\bar{\sigma }}_{r}$	$\bar{r}=0.7$	−1	−1	−1	−1
	$\bar{r}=1.0$	0	0	0	0
${\bar{\sigma }}_{\theta }$	$\bar{r}=0.7$	3.0039	3.0662	3.1908	3.5647
	$\bar{r}=1.0$	1.0739	1.0906	1.1239	1.2239
${\bar{\sigma }}_{z}$	$\bar{r}=0.7$	0.6011	0.6198	0.6572	0.7694
	$\bar{r}=1.0$	0.3187	0.3202	0.3231	0.3321
${\bar{h}}_{z}$	$\bar{r}=0.7$	−0.5698	−0.5875	−0.6229	−0.7293
	$\bar{r}=1.0$	−0.5675	−0.5853	−0.6208	−0.7274

**Table 4:** The extremum values corresponding to different $v_{1}$.

$v_{1}$		0.2	0.3	0.4
$\bar{u}$	$\bar{r}=0.7$	1.8983	1.2608	0.9198
	$\bar{r}=1.0$	1.6490	1.0410	0.7133
${\bar{\sigma }}_{r}$	$\bar{r}=0.7$	−1	−1	−1
	$\bar{r}=1.0$	0	0	0
${\bar{\sigma }}_{\theta }$	$\bar{r}=0.7$	5.0206	3.1908	2.2340
	$\bar{r}=1.0$	1.7002	1.1239	0.8259
${\bar{\sigma }}_{z}$	$\bar{r}=0.7$	1.1041	0.6572	0.4278
	$\bar{r}=1.0$	0.3260	0.3231	0.3163
${\bar{h}}_{z}$	$\bar{r}=0.7$	−1.2549	−0.6229	−0.2753
	$\bar{r}=1.0$	−1.2577	−0.6208	−0.2704

**Table 5:** The assumptions corresponding to different problems.

Case/Refer	Parameters	Load Conditions
1/[32]	*P*_a_ = 1 GPa, *P*_b_ = 0 GPa, *T*_a_ = 0 °C, *T*_b_ = 100 °C, *H*_z_ = 0 A/m	mechanical and thermal loads
2/[33]	*P*_a_ = 1 GPa, *P*_b_ = 0 GPa, *T*_a_ = 0 °C, *T*_b_ = 0 °C, *H*_z_ = 2.23 × 10^9^ A/m	mechanical load and magnetic field
3/[34]	*P*_a_ = 1 GPa, *P*_b_ = 0 GPa, *T*_a_ = 0 °C, *T*_b_ = 0 °C, *H*_z_ = 0 A/m	mechanical load

**Table 6:** The values of material parameters of different cases.

Locations	Case	Theoretical Reference	$\bar{u}$	${\bar{\sigma }}_{r}$	${\bar{\sigma }}_{\theta }$	${\bar{\sigma }}_{z}$	${\bar{h}}_{z}$
$\bar{r}=0.7$	Case 1	This paper	1.3861	−1.0000	3.5505	0.7652	0
		[32]					
$\bar{r}=0.85$		This paper	1.2364	−0.3321	2.1348	0.5317	0
		[32]					
$\bar{r}=1.0$		This paper	1.1423	−0.0350	1.2202	0.3416	0
		[32]					
$\bar{r}=0.7$	Case 2	This paper	1.1740	−1.0000	2.9417	0.5825	−0.5521
		[33]					
$\bar{r}=0.85$		This paper	1.0422	−0.2898	1.8061	0.4549	−0.5504
		[33]					
$\bar{r}=1.0$		This paper	0.9622	0.0000	1.0573	0.3172	−0.5498
		[33]					
$\bar{r}=0.7$	Case 3	This paper	1.4399	−1.0000	3.7051	0.8115	0
		[34]					
$\bar{r}=0.85$		This paper	1.2860	−0.3085	2.2498	0.5824	0
		[34]					
$\bar{r}=1.0$		This paper	1.1886	0.0000	1.3061	0.3918	0
		[34]					

## Data Availability

Data are contained within the article.
